# Supplementary material for: Hyperprogressive Disease during Anti-PD-1 (PDCD1) / PD-L1 (CD274) Therapy: A Systematic Review and Meta-Analysis
Source: Cancers (Basel). 2019 Nov 1;11(11):1699. doi: 10.3390/cancers11111699 (PMC6896059; doi:10.3390/cancers11111699)
Supplement: Supplementary file 1 [file cancers-11-01699-s001.pdf]

Supplementary Materials

Hyperprogressive Disease during Anti-PD-1 (PDCD1) /PD-L1 (CD274) Therapy: A Systematic Review and Meta-Analysis

Index

PRISMA checklist

Full search strategy in PubMed

Table S1. Baseline patient characteristics of included studies

Table S2. Quality assessment of the eligible studies

Table S3. Incidence of hyperprogressive disease

Table S4. Associations between baseline patient characteristics and odds of HPD, subset meta-analysis of cohorts of patients with non-small cell lung cancer

Table S5. Associations between baseline patient characteristics and odds of HPD, subset meta-analysis after excluding two studies including patient data not of our interest

Figure S1. Funnel plot of meta-analysis of association of PD-L1 expression at baseline with hyperprogressive disease

PRISMA checklist

Section/topic	#	Checklist item	Reported on page #
**TITLE**			
Title	1	Identify the report as a systematic review, meta-analysis, or both.	1
**ABSTRACT**			
Structured summary	2	Provide a structured summary including, as applicable: background; objectives; data sources; study eligibility criteria, participants, and interventions; study appraisal and synthesis methods; results; limitations; conclusions and implications of key findings; systematic review registration number.	2
**INTRODUCTION**			
Rationale	3	Describe the rationale for the review in the context of what is already known.	2-3
Objectives	4	Provide an explicit statement of questions being addressed with reference to participants, interventions, comparisons, outcomes, and study design (PICOS).	3
**METHODS**			
Protocol and registration	5	Indicate if a review protocol exists, if and where it can be accessed (e.g., Web address), and, if available, provide registration information including registration number.	NA
Eligibility criteria	6	Specify study characteristics (e.g., PICOS, length of follow-up) and report characteristics (e.g., years considered, language, publication status) used as criteria for eligibility, giving rationale.	14
Information sources	7	Describe all information sources (e.g., databases with dates of coverage, contact with study authors to identify additional studies) in the search and date last searched.	13
Search	8	Present full electronic search strategy for at least one database, including any limits used, such that it could be repeated.	13, appendix
Study selection	9	State the process for selecting studies (i.e., screening, eligibility, included in systematic review, and, if applicable, included in the meta-analysis).	13-14
Data collection process	10	Describe method of data extraction from reports (e.g., piloted forms, independently, in duplicate) and any processes for obtaining and confirming data from investigators.	14
Data items	11	List and define all variables for which data were sought (e.g., PICOS, funding sources) and any assumptions and simplifications made.	14
Risk of bias in individual studies	12	Describe methods used for assessing risk of bias of individual studies (including specification of whether this was done at the study or outcome level), and how this information is to be used in any data synthesis.	14
Summary measures	13	State the principal summary measures (e.g., risk ratio, difference in means).	14
Synthesis of results	14	Describe the methods of handling data and combining results of studies, if done, including measures of consistency (e.g., I^2) for each meta-analysis.	14

Section/topic	#	Checklist item	Reported on page #
Risk of bias across studies	15	Specify any assessment of risk of bias that may affect the cumulative evidence (e.g., publication bias, selective reporting within studies).	14
Additional analyses	16	Describe methods of additional analyses (e.g., sensitivity or subgroup analyses, meta-regression), if done, indicating which were pre-specified.	14
**RESULTS**			
Study selection	17	Give numbers of studies screened, assessed for eligibility, and included in the review, with reasons for exclusions at each stage, ideally with a flow diagram.	3, figure 1
Study characteristics	18	For each study, present characteristics for which data were extracted (e.g., study size, PICOS, follow-up period) and provide the citations.	3, table1-2, table S1
Risk of bias within studies	19	Present data on risk of bias of each study and, if available, any outcome level assessment (see item 12).	3, table S2
Results of individual studies	20	For all outcomes considered (benefits or harms), present, for each study: (a) simple summary data for each intervention group (b) effect estimates and confidence intervals, ideally with a forest plot.	3, figure 2, table S1
Synthesis of results	21	Present results of each meta-analysis done, including confidence intervals and measures of consistency.	3, table 3, figure 2
Risk of bias across studies	22	Present results of any assessment of risk of bias across studies (see Item 15).	3, table 3, figure S1
Additional analysis	23	Give results of additional analyses, if done (e.g., sensitivity or subgroup analyses, meta-regression [see Item 16]).	10, table S4-S5
**DISCUSSION**			
Summary of evidence	24	Summarize the main findings including the strength of evidence for each main outcome; consider their relevance to key groups (e.g., healthcare providers, users, and policy makers).	10-13
Limitations	25	Discuss limitations at study and outcome level (e.g., risk of bias), and at review-level (e.g., incomplete retrieval of identified research, reporting bias).	13
Conclusions	26	Provide a general interpretation of the results in the context of other evidence, and implications for future research.	14
**FUNDING**			
Funding	27	Describe sources of funding for the systematic review and other support (e.g., supply of data); role of funders for the systematic review.	15 (none)

Full search strategy in PubMed

#1. hyperprogress*

#2. hyper-progress*

#3. #1 OR #2

#4. immunotherap*

#5. PD1

#6. PD-1

#7. PD-L1

#8. PDL1

#9. PDCD1

#10. CD274

#11. programmed cell death

#12. programmed death ligand

#13. nivolumab

#14. pembrolizumab

#15. atezolizumab

#16. avelumab

#17. durvalumab

#18. cemiplimab

#19. BMS-936558

#20. BMS-936559

#21. MK-3475

#22. MPDL3280A

#23. MEDI4736

#24. MSB0010718C

#25. immune checkpoint*

#26. checkpoint blockade*

#27. #4 OR #5 OR #6 OR #7 OR #8 OR #9 OR #10 OR #11 OR #12 OR #13 OR #14 OR #15 OR #16 OR #17 OR #18 OR #19 OR #20 OR #21 OR #22 OR #23 OR #24 OR #25 OR #26

#22. #3 AND #27

Table S1. Baseline patient characteristics of included studies

	Champiat, et al. 2017		Kato, et al. 2017		Saada-Bouzid, et al. 2017		Ferrara, et al. 2018		Lo Russo, et al. 2019		Sasaki, et al. 2019		Kanjanapan, et al. 2019		Tunali, et al. 2019		Kim, et al. 2019	
	HPD	non-HPD	HPD	non-HPD	HPD	non-HPD	HPD	non-HPD	HPD	non-HPD	HPD	non-HPD	HPD	non-HPD	HPD	non-HPD	HPD	non-HPD
**Age >= 65**	-	-	2 / 6	36 / 96	-	-	22 / 56	166 / 350	-	-	9 / 13	31 / 49	4 / 12	65 / 170	10 / 15	103 / 172	-	-
**Female sex**	8 / 12	52 / 119	-	-	2 / 10	5 / 24	-	-	17 / 39	46 / 113	1 / 13	16 / 49	10 / 12	73 / 170	5 / 15	77 / 172	20 / 54	52 / 209
**Smoking history**	-	-	-	-	8 / 10	20 / 24	52 / 56	319 / 350	32 / 39	96 / 109	-	-	-	-	14 / 15	135 / 167	30 / 54	138 / 209
**ECOG performance status >= 1**	-	-	-	-	-	-	-	-	-	-	10 / 13	14 / 49	10 / 12	114 / 170	12 / 15	135 / 172	-	-
**ECOG performance status >= 2**	-	-	-	-	-	-	7 / 56	39 / 350	5 / 39	6 / 113	-	-	-	-	-	-	40 / 54	163 / 209
**RMH prognostic score >= 2**	7 / 12	42 / 119	-	-	-	-	-	-	-	-	-	-	6 / 12	68 / 170	11 / 15	48 / 155	27 / 43	32 / 109
**Neutrophil-to-lymphocyte ratio <= 3**	-	-	-	-	-	-	21 / 31	174 / 254	-	-	-	-	-	-	-	-	29 / 54	121 / 209
**Serum lactate dehydrogenase > upper limit of normal**	-	-	-	-	-	-	10 / 27	59 / 192	-	-	-	-	-	-	-	-	27 / 43	44 / 109
**Number of metastatic sites > 2**	6 / 12	67 / 119	-	-	-	-	35 / 56	149 / 350	24 / 39	62 / 113	7 / 13	14 / 49	-	-	10 / 15	95 / 172	35 / 54	94 / 209
**Liver metastasis**	-	-	-	-	-	-	-	-	13 / 39	16 / 113	10 / 13	20 / 49	-	-	5 / 15	34 / 172	16 / 54	20 / 209
**PD-L1 positive**	2 / 3	30 / 32	-	-	-	-	5 / 12	73 / 105	10 / 22	35 / 68	1 / 13	11 / 49	-	-	-	-	25 / 39	127 / 182
**PD-1 inhibitor vs PD-L1 inhibitor**	7 / 12	71 / 119	-	-	5 / 10	18 / 24	52 / 56	325 / 350	29 / 39	71 / 112	-	-	-	-	8 / 11	50 / 99	51 / 54	195 / 209
**Combination therapy vs monotherapy**	-	-	-	-	-	-	2 / 56	24 / 350	5 / 39	2 / 112	-	-	4 / 12	32 / 170	-	-	-	-
**Previous treatment lines > 2**	-	-	-	-	-	-	24 / 56	164 / 350	17 / 39	49 / 113	9 / 13	25 / 49	-	-	-	-	29 / 54	88 / 209
**Previous chemotherapy**	9 / 12	79 / 119	-	-	-	-	50 / 56	307 / 350	-	-	-	-	-	-	9 / 15	67 / 172	38 / 54	157 / 209
**Previous radiotherapy**	6 / 12	53 / 119	-	-	10 / 10	23 / 24	0 / 56	17 / 350	-	-	1 / 13	3 / 49	-	-	1 / 15	41 / 172	-	-
**Previous targeted therapy**	8 / 12	65 / 119	-	-	-	-	5 / 56	44 / 350	-	-	-	-	-	-	4 / 15	32 / 172	10 / 54	21 / 209
**Previous immunotherapy**	3 / 12	17 / 119	-	-	-	-	1 / 56	2 / 350	-	-	-	-	-	-	-	-	-	-
**Previous corticosteroid**	2 / 12	6 / 119	-	-	-	-	-	-	-	-	-	-	-	-	3 / 15	28 / 172	-	-
**EGFR mutation**	-	-	2 / 6	6 / 96	-	-	0 / 36	16 / 233	2 / 31	5 / 96	-	-	-	-	0 / 9	20 / 125	8 / 54	23 / 209
**KRAS mutation**	-	-	-	-	-	-	-	-	2 / 39	13 / 113	-	-	-	-	2 / 7	26 / 84	-	-
**ALK rearrangement**	-	-	-	-	-	-	1 / 36	3 / 233	1 / 31	0 / 96	-	-	-	-	-	-	1 / 54	2 / 209
**Squamous histology**	-	-	-	-	-	-	14 / 56	98 / 350	13 / 39	28 / 113	-	-	-	-	4 / 15	52 / 172	12 / 54	70 / 209

Abbreviations: ECOG, Eastern Cooperative Oncology Group; HPD, hyperprogressive disease; PD-1, programmed cell death protein 1; PD-L1, programmed death-ligand 1; RMH, Royal Marsden Hospital

Table S2. Quality assessment of the eligible studies

	1. Representativeness of the exposed cohort	2. Selection of the non exposed cohort	3. Ascertainment of exposure	4. Outcome (HPD), by definition, was not present at start of study	5. Adjustment for at least one major pathoclinical variable	6. Adjustment for more than one major pathoclinical variables	7. Outcome (HPD) measurement	8. Follow-up at one imaging after the treatment initiation and/or two months after treatment initiation	9. Adequacy of follow up of cohorts. Not eligible, because all studies retrospectively examined only the patient data in which HPD ascertainment were possible	Total score
**Champiat, et al. 2017**	1	1	1	1	1	0	1	1	0	7
**Kato, et al. 2017**	1	1	1	1	1	1	1	1	0	8
**Saada-Bouzid, et al. 2017**	1	1	1	1	0	0	1	1	0	6
**Ferrara, et al. 2018**	1	1	1	1	0	0	1	1	0	6
**Lo Russo, et al. 2019**	1	1	1	1	0	0	1	1	0	6
**Sasaki, et al. 2019**	1	1	1	1	0	0	1	1	0	6
**Kanjanapan, et al. 2019**	1	1	1	1	0	0	1	1	0	6
**Tunali, et al. 2019**	1	1	1	1	0	0	1	1	0	6
**Kim, et al. 2019**	1	1	1	1	0	0	1	1	0	6

Table S3. Incidence of hyperprogressive disease

Underlying malignancy	City, country (institution)	Definition of HPD	HPD Incidence
Advanced gastric cancer[1]	Japan	• TGKpost/TGKpre >= 2 and > 50% increase in tumor burden	21% (13/62)
Triple-negative breast cancer[2]	Toronto, Canada	• TGRpost/TGRpre >=2	10% (4/40)
Hepatocellular carcinoma[3]	Austria and Germany	• RECIST-defined PD at first evaluation and TGRpost - TGRpre > 50%	8% (4/52)
High grade glioma[4]	New York, USA	• TGRpost/TGRpre >=2	1% (1/102)
High grade glioma[5]	New York, USA	• TGRpost/TGRpre >=2	28% (7/25)
HNSCC[6]	Italy	• RECIST-defined PD at first evaluation and TGKpost/TGKpre >= 2	8% (7/88)
HNSCC[7]	Barcelona, Spain	• TGRpost/TGRpre >=2	4% (2/46)
HNSCC[8]	France	• TGRpost/TGRpre >=2	29% (10/34)
HNSCC[9]	Greece	• TGKpost/TGKpre >= 2 or disease-related rapid clinical deterioration	26% (16/62)
Melanoma[10]	China	• RECIST-defined PD at first evaluation and TGRpost/TGRpre >=2	6% (5/90)
Melanoma[11]	France	• Progression/death within 3 months with normal initial LDH and ECOG at baseline, and either ECOG increased from 0 to 3-4, either LDH increased from normal to elevated or both	10% (82/793)
NSCLC[12]	Korea (Yonsei University)	• RECIST-defined PD at first evaluation and TGRpost/TGRpre >=2 or TGKpost/TGKpre >= 2	21% (54/263)
NSCLC[13]	Florida, USA (Moffitt Cancer Center)	• RECIST-defined PD at first evaluation and TGRpost/TGRpre >=2 and TTF < 2 months	8% (15/187)
NSCLC[14]	Italy (Thoracic Unit of the Medical Oncology Department at the Istituto Nazionale dei Tumori)	• Fulfilling at least 3 of the following 5 criteria: 1) TTF < 2 months, 2) > 50% increase in the sum of target lesions major diameters between baseline and first radiologic evaluation, 3) appearance of at least two new lesions in an organ already involved between baseline and first radiologic evaluation, 4) spread of the disease to a new organ between baseline and first radiologic evaluation, 5) ECOG >= 2 during the first 2 months of treatment	26% (39/152)
NSCLC[15]	France	• RECIST-defined PD at first evaluation and TGRpost - TGRpre > 50%	14% (56/406)
NSCLC[16]	Korea (St. Mary Hospital, the Catholic University of Korea)	• TGKpost/TGKpre >=2 and TTF < 2 months	11% (25/231)
NSCLC[17]	Italy	• TTF <= 2 months and >= 50% increase in tumor burden	25% (5/20)
NSCLC[18]	Korea (Samsung Medical Center)	Criteria using TGRpre and TGRpost. Details NA.	17% (37/220)
NSCLC[19]	Italy	• > 50% increase in tumor burden	2% (1/46)
NSCLC[20]	Spain	• TGRpost/TGRpre >=2	30% (12/40)
Renal cell carcinoma[21]	Korea	• TGRpost/TGRpre >= 2 and > 50% increase in tumor burden, or development of extensive new lesions	1% (1/102)
Renal cell carcinoma [22]	Spain	• TTF < 2 months and minimum increase in measurable lesions of 10 mm plus: 1) increase of >= 40% in target tumor burden compared to baseline or 2) increase of >= 20% plus the appearance of multiple new lesions.	7% (2/29)
Renal cell carcinoma [23]	France	• TGRpost - TGRpre > 50%	3% (1/39)
Urothelial cell carcinoma[21]	Korea	• TGRpost/TGRpre >= 2 and > 50% increase in tumor burden, or development of extensive new lesions	12% (12/101)
Urothelial cell carcinoma [22]	Spain	• TTF < 2 months and minimum increase in measurable lesions of 10 mm plus: 1) increase of >= 40% in target tumor burden compared to baseline or 2) increase of >= 20% plus the appearance of multiple new lesions.	12% (7/59)

References of table

1. Kamada T.; Togashi Y.; Tay C.; Ha D.; Sasaki A.; Nakamura Y.; Sato E.; Fukuoka S.; Tada Y.; Tanaka A., et al. PD-1(+) regulatory T cells amplified by PD-1 blockade promote hyperprogression of cancer. Proc Natl Acad Sci U S A 2019, 116, 9999-10008.
2. Tan T. J. Y.; Cescon D. W.; Wang L.; Amir E.; Vieira D.; Zammit K.; Warr D.; Elser C.; Butler M. O.; Razak A. R. A., et al. Hyperprogressive disease in advanced triple-negative breast cancer (aTNBC) treated with immunotherapy (IO). J Clin Oncol 2019, 37, 1086-1086.
3. Scheiner B.; Kirstein M. M.; Hucke F.; Finkelmeier F.; Schulze K.; von Felden J.; Koch S.; Schwabl P.; Hinrichs J. B.; Waneck F., et al. Programmed cell death protein-1 (PD-1)-targeted immunotherapy in advanced hepatocellular carcinoma: efficacy and safety data from an international multicentre real-world cohort. Aliment Pharmacol Ther 2019, 49, 1323-1333.
4. Donovan L.; Schulte J.; Kreisl T.; Welch M.; Lassman A. B.; Iwamoto F. MDM2/4 AMPLIFICATION AND RISK OF HYPERPROGRESSION IN HIGH-GRADE GLIOMAS TREATED WITH CHECKPOINT INHIBITORS. Neuro Oncol 2018, 20, 158-158.
5. Donovan L.; Gedailovich S.; Joanta-Gomez A.; Schulte J.; Kreisl T. N.; Lassman A. B.; Welch M. R.; Haggiagi A.; Iwamoto F. M. Hyperprogressive disease in patients with recurrent high grade gliomas treated with immune checkpoint inhibitors or other therapies. J Clin Oncol 2019, 37, e13575-e13575.
6. Alfieri S.; Ferrara R.; Calareso G.; Cavalieri S.; Platini F.; Mancinelli M.; Resteghini C.; Orlandi E.; Iacovelli N. A.; Ferella L., et al. Hyperprogressive disease (HPD) in head and neck squamous cell carcinoma (HNSCC) patients treated with immune checkpoint inhibitors (ICI). J Clin Oncol 2019, 37, 6029-6029.
7. Ortega Franco A.; Plana M.; Braña I.; Taberna Sanz M.; Oliva Bernal M.; Vázquez S.; Domenech Vinyolas M.; Berenguer G.; Vilajosana E.; Bergamino M., et al. Does hyper-progression exist among head and neck cancer patients treated with immunotherapy? Ann Oncol 2017, 28, v379.
8. Saada-Bouzid E.; Defaucheux C.; Karabajakian A.; Coloma V. P.; Servois V.; Paoletti X.; Even C.; Fayette J.; Guigay J.; Loirat D., et al. Hyperprogression during anti-PD-1/PD-L1 therapy in patients with recurrent and/or metastatic head and neck squamous cell carcinoma. Ann Oncol 2017, 28, 1605-1611.
9. Economopoulou P.; Spathas N. S.; Papaxoinis G.; Anastasiou M.; Gkotzamanidou M.; Kotsantis I.; Gavrielatou N.; Oikonomopoulos N.; Kirodimos E.; Vagia E. M., et al. Clinical implications of hyperprogression with immune checkpoint inhibitors in patients with head and neck squamous cell carcinoma (HNSCC). J Clin Oncol 2019, 37, 6034-6034.
10. Tang B.; Chi Z.; Sheng X.; Si L.; Cui C.; Kong Y.; Yan X.; Li S.; Mao L.; LIAN B., et al. Tumor growth rate as an early indicator of the efficacy of anti-PD-1 immunotherapy in advanced melanoma. J Clin Oncol 2019, 37, e21050-e21050.
11. Colle E.; Dalle S.; Mortier L.; Guillot B.; Dutriaux C.; Leccia M. T.; Dalac S.; Legoupil D.; Quatrebarbes J. D.; Montaudié H., et al. Progression and hyperprogression after anti-PD1 therapy for unresectable stage III or IV melanoma patients. J Clin Oncol 2019, 37, e21021-e21021.
12. Kim C. G.; Kim K. H.; Pyo K. H.; Xin C. F.; Hong M. H.; Ahn B. C.; Kim Y.; Choi S. J.; Yoon H. I.; Lee J. G., et al. Hyperprogressive disease during PD-1/PD-L1 blockade in patients with non-small-cell lung cancer. Ann Oncol 2019.
13. Tunali I.; Gray J. E.; Qi J.; Abdalah M.; Jeong D. K.; Guvenis A.; Gillies R. J.; Schabath M. B. Novel clinical and radiomic predictors of rapid disease progression phenotypes among lung cancer patients treated with immunotherapy: An early report. Lung Cancer 2019, 129, 75-79.
14. Lo Russo G.; Moro M.; Sommariva M.; Cancila V.; Boeri M.; Centonze G.; Ferro S.; Ganzinelli M.; Gasparini P.; Huber V., et al. Antibody-Fc/FcR Interaction on Macrophages as a Mechanism for Hyperprogressive Disease in Non-small Cell Lung Cancer Subsequent to PD-1/PD-L1 Blockade. Clin Cancer Res 2019, 25, 989-999.
15. Ferrara R.; Mezquita L.; Texier M.; Lahmar J.; Audigier-Valette C.; Tessonnier L.; Mazieres J.; Zalcman G.; Brosseau S.; Le Moulec S., et al. Hyperprogressive Disease in Patients With Advanced Non-Small Cell Lung Cancer Treated With PD-1/PD-L1 Inhibitors or With Single-Agent Chemotherapy. JAMA Oncol 2018, 4, 1543-1552.
16. Kim S.; Kang J.-H.; Chun S. H.; Kim J. Clinical implication of inflammation-based serologic biomarkers and tissue biomarkers on hyperprogression in NSCLC patients receiving immune checkpoint blockers in real world. J Clin Oncol 2019, 37, e20633-e20633.
17. Giusti R.; Mazzotta M.; Filetti M.; Marinelli D.; Napoli A. D.; Scarpino S.; Scafetta G.; Mei M.; Vecchione A.; Ruco L., et al. CDKN2A/B gene loss and MDM2 alteration as a potential molecular signature for hyperprogressive disease in advanced NSCLC: A next-generation-sequencing approach. J Clin Oncol 2019, 37, e20628-e20628.
18. Kim Y.; Kim C. H.; Kim H. S.; Sun J. M.; Ahn J. S.; Ahn M. J.; Lee S. H.; Lee H. Y.; Park K. Hyperprogression after immunotherapy: Clinical implication and genomic alterations in advanced non-small cell lung cancer patients (NSCLC). J Clin Oncol 2018, 36.
19. Perna M.; Scotti V.; Muntoni C.; Moroni C.; Giannelli F.; Cozzi D.; Mazzoni F.; Bonti V.; Lavacchi D.; Livi L. Clinico-radiological pattern of response to nivolumab in stage IV NSCL: A real life experience over two years. J Thorac Oncol 2018, 13, S110.
20. Simões da Rocha P. F.; Ripoll E.; Corbera A.; Hardy-Werbin M.; Fernandez-Alarza A. F.; Orrillo M.; Taus Á.; Arriola E. Radiological identification of rapid progressions in advanced NSCLC patients treated with nivolumab. Ann Oncol 2018, 29.
21. Hwang I.; Park I.; Yoon S. K.; Lee J. L. Hyperprogressive disease (HPD) in genitourinary (GU) cancer patients treated with PD-1/PD-L1 inhibitors. J Clin Oncol 2019, 37.
22. Suarez C.; Morales-Barrera R.; Garcia-Ruiz A.; Gonzalez M.; Ligero M.; Valverde C.; Serrano C.; Mateo J.; Perez-Lopez R.; Carles J. Hyperprogressive disease in patients with metastatic genitourinary tumors treated with immune checkpoint inhibitors. J Clin Oncol 2019, 37.
23. Mazza C.; Arfi-Rouche J.; Koscielny S.; Caramella C.; Lahmar J.; Escudier B. J.; Albiges L. Effect of nivolumab on tumor growth rate (TGR) in metastatic renal cell carcinoma (mRCC). J Clin Oncol 2017, 35.

Table S4. Associations between baseline patient characteristics and odds of HPD, subset meta-analysis of cohorts of patients with non-small cell lung cancer

Baseline patient characteristics	Number of study estimates	Number of HPD/non-HPD patients	Random-effects summary estimate, odds ratio and 95% confidence interval*	P value*	I^2 (%)	95% prediction interval†	Egger p value†
**Age >= 65**	2	71/522	0.82 (0.49 to 1.36)	0.44	0%	NA	NA
**Female sex**	3	108/494	1.23 (0.72 to 2.10)	0.46	28%	0.01 to 142.89	0.19
**Smoking history**	4	164/835	0.80 (0.49 to 1.30)	0.36	7%	0.23 to 2.78	0.14
**ECOG performance status >= 1**	3	149/672	1.14 (0.63 to 2.04)	0.67	24%	0.01 to 175.13	0.0075
**Neutrophil-to-lymphocyte ratio <= 3**	2	85/463	0.89 (0.55 to 1.43)	0.62	0%	NA	NA
**Serum lactate dehydrogenase > upper normal limit**	2	70/301	**1.89 (1.02 to 3.49)**	**0.043**	19%	NA	NA
**Number of metastatic sites > 2**	4	164/844	**1.94 (1.36 to 2.75)**	**0.00022**	0%	0.90 to 4.17	0.40
**Liver metastases**	3	108/494	**3.17 (1.92 to 5.24)**	**0.0000066**	0%	0.12 to 82.37	0.11
**RMH prognostic score >= 2**	2	58/264	**4.56 (2.42 to 8.56)**	**0.0000025**	0%	NA	NA
**PD-L1 positive**	3	73/355	0.66 (0.39 to 1.11)	0.12	0%	0.02 to 19.67	0.38
**PD-1 inhibitor vs PD-L1 inhibitor**	4	160/770	1.50 (0.87 to 2.56)	0.14	0%	0.46 to 4.87	0.95
**Combination therapy vs monotherapy**	2	95/462	1.95 (0.13 to 29.70)	0.63	83%	NA	NA
**Previous treatment lines > 2**	3	149/672	1.11 (0.75 to 1.64)	0.59	13%	0.06 to 21.69	0.99
**Previous chemotherapy**	3	125/731	1.15 (0.63 to 2.09)	0.64	31%	0.01 to 258.04	0.16
**Previous radiotherapy**	2	71/522	0.21 (0.04 to 1.08)	0.062	0%	NA	NA
**Previous targeted therapy**	3	125/731	1.33 (0.67 to 2.63)	0.42	32%	0.00 to 670.11	0.82
***EGFR* mutation**	4	130/663	1.09 (0.53 to 2.24)	0.82	0%	0.22 to 5.30	0.089
***KRAS* mutation**	2	46/197	0.59 (0.19 to 1.83)	0.36	0%	NA	NA
***ALK* rearrangement**	3	121/538	2.86 (0.65 to 12.52)	0.16	0%	0.00 to 41535.21	0.15
**Squamous histology**	4	164/844	0.87 (0.58 to 1.31)	0.50	10%	0.30 to 2.53	0.82

*Statistically significant associations are shown in bold.
†Not available for meta-analysis of two studies
All statistical tests are two-sided.
Abbreviations: ECOG, Eastern Cooperative Oncology Group; HPD, hyperprogressive disease; NA, not available; PD-1, programmed cell death protein 1; PD-L1, programmed death-ligand 1; RMH, Royal Marsden Hospital

Table S5. Associations between baseline patient characteristics and odds of HPD, subset meta-analysis after excluding two studies including patient data not of our interest

Baseline patient characteristics	Number of study estimates	Number of HPD/non-HPD patients	Random-effects summary estimate, odds ratio and 95% confidence interval*	P value*	I^2 (%)	95% prediction interval†	Egger p value
**Age >= 65**	4	90/667	0.87 (0.55 to 1.37)	0.54	0%	0.32 to 2.38	0.29
**Female sex**	5	131/567	1.03 (0.57 to 1.84)	0.93	35%	0.22 to 4.75	0.087
**Smoking history**	5	174/859	0.78 (0.50 to 1.22)	0.28	0%	0.38 to 1.61	0.18
**ECOG performance status >= 2**	2	28/221	2.96 (0.41 to 21.61)	0.28	76%	NA	NA
**ECOG performance status >= 1**	3	149/672	1.14 (0.63 to 2.04)	0.67	24%	0.01 to 175.13	0.0075
**Neutrophil-to-lymphocyte ratio <= 3**	2	85/463	0.89 (0.55 to 1.43)	0.62	0%	NA	NA
**Serum lactate dehydrogenase > upper normal limit**	2	70/301	**1.89 (1.02 to 3.49)**	**0.043**	19%	NA	NA
**Number of metastatic sites > 2**	5	177/893	**1.99 (1.42 to 2.79)**	**0.000061**	0%	1.15 to 3.45	0.90
**Liver metastases**	4	121/543	**3.33 (2.07 to 5.34)**	**0.00000064**	0%	1.18 to 9.4	0.86
**RMH prognostic score >= 2**	2	58/264	**4.56 (2.42 to 8.56)**	**0.0000025**	0%	NA	NA
**PD-L1 positive**	4	86/404	0.63 (0.38 to 1.04)	0.073	0%	0.21 to 1.92	0.20
**PD-1 inhibitor vs PD-L1 inhibitor**	5	170/794	1.25 (0.72 to 2.17)	0.42	12%	0.40 to 3.91	0.42
**Combination therapy vs monotherapy**	2	95/462	1.95 (0.13 to 29.70)	0.63	83%	NA	NA
**Previous treatment lines > 2**	4	162/721	1.17 (0.81 to 1.68)	0.40	7%	0.47 to 2.91	0.49
**Previous chemotherapy**	3	125/731	1.15 (0.63 to 2.09)	0.64	31%	0.01 to 258.04	0.16
**Previous radiotherapy**	4	94/595	0.46 (0.13 to 1.60)	0.22	0%	0.03 to 7.15	0.68
**Previous targeted therapy**	3	125/731	1.33 (0.67 to 2.63)	0.42	32%	0.00 to 670.11	0.82
**NSCLC** / ***EGFR* mutation**	5	136/759	1.29 (0.48 to 3.52)	0.61	37%	0.09 to 19.35	0.57
***KRAS* mutation**	2	46/197	0.59 (0.19 to 1.83)	0.36	0%	NA	NA
***ALK* rearrangement**	3	121/538	2.86 (0.65 to 12.52)	0.16	0%	0.00 to 41535.21	0.15
**Squamous histology**	4	164/844	0.87 (0.58 to 1.31)	0.50	10%	0.30 to 2.53	0.82

*Statistically significant associations are shown in bold.
†Not available for meta-analysis of two studies
All statistical tests are two-sided.
Abbreviations: ECOG, Eastern Cooperative Oncology Group; HPD, hyperprogressive disease; NA, not available; NSCLC, non-small cell lung cancer; PD-1, programmed cell death protein 1; PD-L1, programmed death-ligand 1; RMH, Royal Marsden Hospital

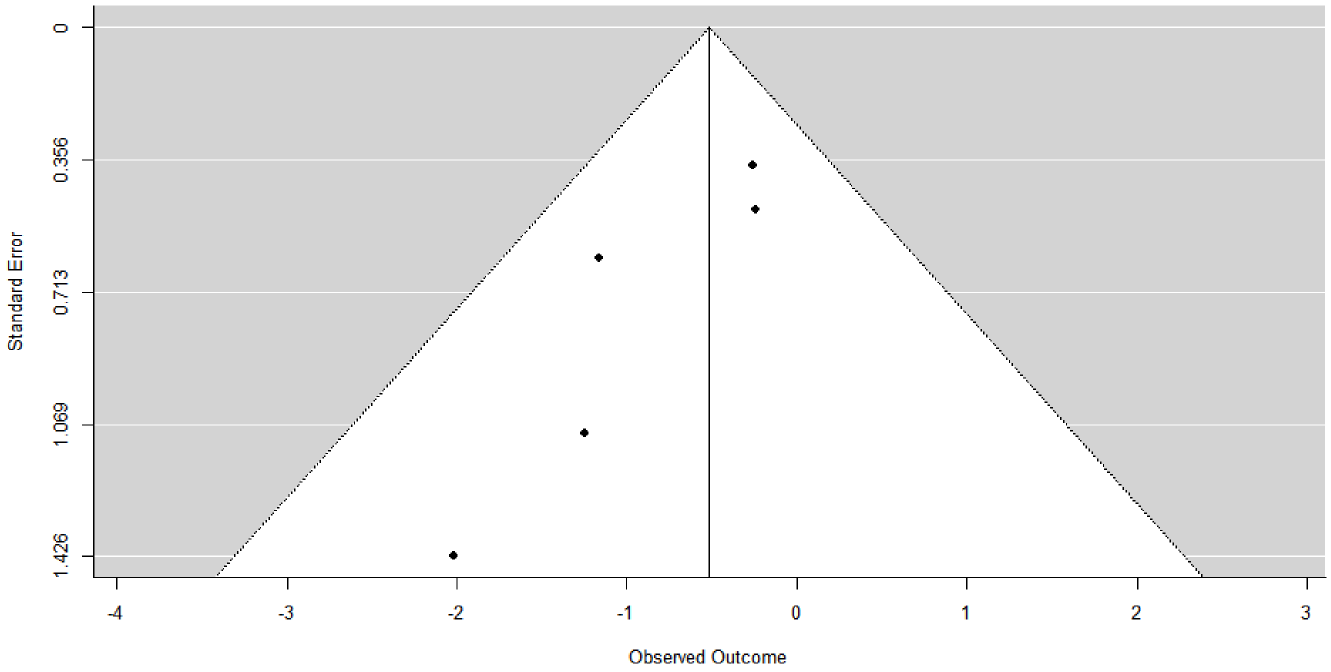

Figure S1. Funnel plot of meta-analysis of association of PD-L1 expression at baseline with hyperprogressive disease
